# Culture dependent and independent analysis of bacterial communities associated with commercial salad leaf vegetables

**DOI:** 10.1186/1471-2180-13-274

**Published:** 2013-12-01

**Authors:** Colin R Jackson, Kevin C Randolph, Shelly L Osborn, Heather L Tyler

**Affiliations:** 1 Department of Biology, The University of Mississippi, University 38677, USA

**Keywords:** Bacterial endophytes, Salad produce, Food-borne bacteria, Pyrosequencing

## Abstract

**Background:**

Plants harbor a diverse bacterial community, both as epiphytes on the plant surface and as endophytes within plant tissue. While some plant-associated bacteria act as plant pathogens or promote plant growth, others may be human pathogens. The aim of the current study was to determine the bacterial community composition of organic and conventionally grown leafy salad vegetables at the point of consumption using both culture-dependent and culture-independent methods.

**Results:**

Total culturable bacteria on salad vegetables ranged from 8.0 × 10^3^ to 5.5 × 10^8^ CFU g^-1^. The number of culturable endophytic bacteria from surface sterilized plants was significantly lower, ranging from 2.2 × 10^3^ to 5.8 × 10^5^ CFU g^-1^. Cultured isolates belonged to six major bacterial phyla, and included representatives of *Pseudomonas*, *Pantoea*, *Chryseobacterium*, and *Flavobacterium*. Eleven different phyla and subphyla were identified by culture-independent pyrosequencing, with Gammaproteobacteria, Betaproteobacteria, and Bacteroidetes being the most dominant lineages. Other bacterial lineages identified (e.g. Firmicutes, Alphaproteobacteria, Acidobacteria, and Actinobacteria) typically represented less than 1% of sequences obtained. At the genus level, sequences classified as *Pseudomonas* were identified in all samples and this was often the most prevalent genus. *Ralstonia* sequences made up a greater portion of the community in surface sterilized than non-surface sterilized samples, indicating that it was largely endophytic, while *Acinetobacter* sequences appeared to be primarily associated with the leaf surface. Analysis of molecular variance indicated there were no significant differences in bacterial community composition between organic versus conventionally grown, or surface-sterilized versus non-sterilized leaf vegetables. While culture-independent pyrosequencing identified significantly more bacterial taxa, the dominant taxa from pyrosequence data were also detected by traditional culture-dependent methods.

**Conclusions:**

The use of pyrosequencing allowed for the identification of low abundance bacteria in leaf salad vegetables not detected by culture-dependent methods. The presence of a range of bacterial populations as endophytes presents an interesting phenomenon as these microorganisms cannot be removed by washing and are thus ingested during salad consumption.

## Background

Just as animals harbor a complex microbiome, plants are increasingly being recognized as having a diverse bacterial community associated with them [1-3]. Bacterial communities associated with the aboveground portion of plants can be found on both the leaf surface (the phyllosphere) and within plant tissues as endophytes. These endophytic bacteria are present within both vascular tissue and intercellular spaces, can be diverse, and likely originate from soil around plant roots or from the leaf surface [1,4,5]. Virtually every plant studied has yielded isolates of endophytic bacteria, suggesting that all plant species are probably colonized by some endophytic populations [1]. While some plant-associated bacteria may be plant pathogens, others may act as commensals or symbionts, potentially playing roles in plant growth or disease resistance [6,7]. Some plant associated bacteria may also be human pathogens, and pathogenic bacteria can exist as endophytes having entered the host plant through the root system or via wounds, lenticels, and stomata [8-10]. Such endophytic pathogen populations have been linked to food-borne disease outbreaks involving bagged spinach and lettuce [11,12].

Most studies identifying human pathogens in plants have been field or greenhouse studies, or have sampled freshly harvested crops [13]. Few studies have examined the presence of endophytes or surface associated bacteria from the perspective of human consumption, by sampling minimally processed vegetables such as ready-to-eat salad produce. Similarly, few studies have focused on the entire endophyte community, rather than just potential pathogens, even though native endophytic bacterial populations could potentially serve as competitors to such organisms [14,15]. A more diverse community of endophytes has been linked to reduced levels of internal *Salmonella* colonization in lettuce [16], likely because a higher diversity of endophytes means that there is a greater chance of bacteria that are antagonistic to pathogen colonization being present. Thus, determining the composition of endophytic communities in pre-packaged salad produce could provide insights into outbreaks of produce-related illness and lead to the development of more powerful predictive tools for food-borne disease outbreaks.

Endophytic and phyllosphere bacteria have typically been characterized and enumerated using traditional culture based approaches, although such methods are highly dependent on the medium used for isolation and the incubation conditions [17]. In contrast, culture-independent 16S rRNA-based methods can detect unculturable bacterial colonizers of plants, as well as those bacteria that are in such low abundance or grow so slowly that they are missed by traditional culture based protocols. Next generation pyrosequencing of 16S rRNA genes provides a high resolution approach to assess these plant-associated communities and is beginning to be applied to studies of the phyllosphere in environmental systems [18] or to the surface of produce [19]. However, such studies have generally just characterized the composition of the bacterial community on the leaf surface rather than the entire plant-associated bacterial community, which would include endophytic populations.

The aim of the current study was to determine the bacterial community composition of leafy salad vegetables at the point of consumption. To that end, ten types of commercial, ready-to-eat salad leaf vegetables were sampled, representing five different vegetables each of organically grown and conventionally grown varieties. Culturable bacteria were enumerated and identified, and the total plant-associated and endophytic bacterial community structure was analysed using culture-independent next generation pyrosequencing of 16S rRNA gene amplicons.

## Results and discussion

### Culturable bacterial plate counts

**Figure 1:**
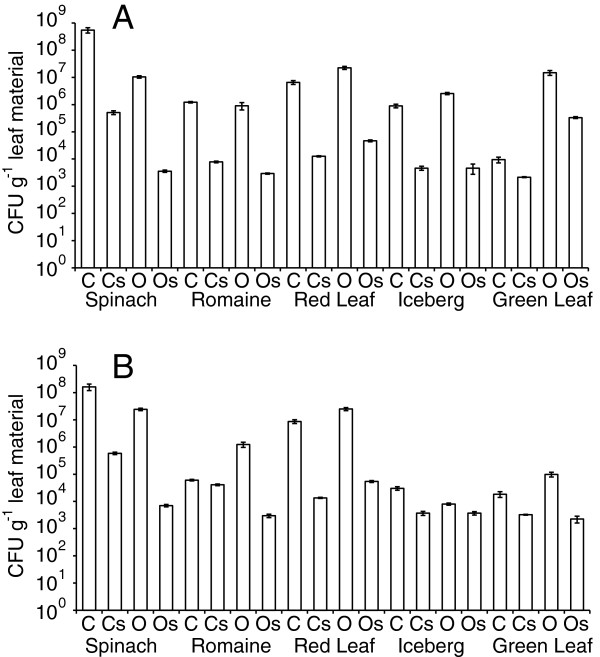
**Viable counts of culturable bacteria obtained from leafy salad vegetables.** Samples were plated on TSA **(A)** and R2A **(B)** media and are baby spinach, romaine lettuce, red leaf lettuce, iceberg lettuce, and green leaf lettuce of conventionally (C) and organically (O) grown varieties. Subsamples of each type were also subjected to surface sterilization (s) prior to processing. Counts represent means (+/− SE) of three analytical replicate plates per sample.

Samples of ten different leafy salad vegetables (organic and conventionally grown romaine lettuce, baby spinach, green leaf lettuce, iceberg lettuce, and red leaf lettuce) obtained from a grocery store were analysed by culture-dependent (plating) and independent (16S rRNA gene sequencing) approaches. Each sample was analysed in an intact, non-surface sterilized form, and also following surface-sterilization. Plates from non-surface sterilized samples yielded substantial numbers of culturable bacteria associated with leafy salad vegetables, ranging from 8.0 × 10^3^ CFUs g^-1^ for the organic iceberg lettuce sample on R2A agar to 5.5 × 10^8^ CFU g^-1^ for the baby spinach sample on TSA. Plate counts for surface-sterilized samples were consistently lower than non-sterilized samples (Figure 1), a difference that was statistically significant (pairwise t-test, p < 0.05). For most samples, surface sterilization reduced plate counts by at least two orders or magnitude, regardless of the growth medium used. However, the reduction in counts following surface sterilization varied by sample, with the surface sterilized sample of organic baby spinach having just 0.03% of the CFUs of the unsterilized sample, while the surface sterilized sample of conventional romaine lettuce still yielded counts that were 67% of the non-sterilized subsample. Other samples that still showed appreciable counts (> 5% of non-sterilized numbers) following surface sterilization included the conventional and organic samples of iceberg lettuce (on R2A media), and the conventional sample of green leaf lettuce (Figure 1), suggesting that these samples had large endophytic bacterial populations. All surface sterilized samples still harbored substantial numbers of bacteria, with colony counts ranging from 2.2 × 10^3^ (the green leaf lettuce sample on TSA) to 5.8 × 10^5^ (the baby spinach sample on R2A agar) CFUs g^-1^ leaf material, a range typical of the culturable population densities of endophytic bacteria [20]. While counts for individual samples differed slightly when grown on TSA or R2A agar, there was no consistent pattern in terms of one growth medium yielding more colonies than the other (pairwise t-test, p = 0.33), and counts on the two media were highly correlated (R = 0.98). The conventionally and organically grown samples of baby spinach and red leaf lettuce yielded the highest CFUs, but there was no pattern of organically grown produce always giving higher or lower microbial counts than the equivalent conventionally grown variety (pairwise t-test, p = 0.27; Figure 1).

### Identity of cultured isolates

Across all samples, a total of 151 isolates were obtained, which corresponded to 31 different bacterial taxa, representing six different major phyla of bacteria (Table 1). Four of these taxa were species of *Pseudomonas* (members of the *P. fluorescens*, *P. chlororaphis*, and *P. syringae* groups, along with an unidentified species) and this genus was the most ubiquitous, being isolated from every sample other than the surface sterilized organic and conventional iceberg lettuce. Given that the particular pseudomonads obtained are recognized as being endophytes or plant pathogens [5], their presence in a wide variety of salad vegetables is not surprising. Other taxa that were isolated from a variety of samples included species of *Pantoea* (eight samples), *Chryseobacterium* (six samples), and *Flavobacterium* (six samples). None of these showed distinct patterns in their distribution (e.g. being isolated only from surface-sterilized samples), and as with *Pseudomonas*, these genera contain species that have been detected in or on plants. The isolation of *Pantoea* species from at least one sample of all of the salad vegetable types (baby spinach, romaine lettuce, red leaf lettuce, iceberg lettuce and green leaf lettuce) is interesting, as while species of *Pantoea* are typically plant commensals or even pathogens, some strains can also be opportunistic human pathogens [21-23].

**Table 1:** Bacterial isolates obtained from store-bought leafy salad vegetables using TSA or R2A media

**Species/Genus (Phylum)**	**Baby spinach**				**Romaine lettuce**				**Red leaf lettuce**				**Iceberg lettuce**				**Green leaf lettuce**			
	**C**	**Cs**	**O**	**Os**	**C**	**Cs**	**O**	**Os**	**C**	**Cs**	**O**	**Os**	**C**	**Cs**	**O**	**Os**	**C**	**Cs**	**O**	**Os**
*Acinetobacter* (Gammaproteobacteria)	-	-	+	-	-	-	-	-	-	-	-	-	+	-	-	-	-	-	-	-
*Aeromicrobium* (Actinobacteria)	-	-	-	-	-	-	-	-	-	-	-	-	-	-	-	-	-	+	-	-
*Agrobacterium* (Alphaproteobacteria)	-	-	-	-	-	-	-	-	-	-	-	-	-	+	-	-		+	-	-
*Arthrobacter* (Actinobacteria)	-	-	-	-	-	-	+	+	-	-	-	-	-	-	-	-	+	+	-	-
*Bacillus flexus* (Firmicutes)	-	-	-	-	-	-	+	+	-	-	-	-	-	-	-	-	-	-	-	-
*Chryseobacterium* (Bacteroidetes)	-	-	-	-	-	-	-	-	-	-	-	-	-	+	-	+	+	+	+	+
*Curtobacterium* (Actinobacteria)	-	-	-	+	-	-	-	-	+	-	-	+	-	-	-	-	-	-	-	-
*Devosia* (Alphaproteobacteria)	-	-	-	-	-	-	-	-	-	-	-	+	-	-	-	-	-	-	-	-
*Erwinia* (Gammaproteobacteria)	-	-	-	-	-	-	-	-	-	-	-	-	-	+	+	+	-	-	-	-
*Flavobacterium* (Bacteroidetes)	+	+	+	-	-	+	-	-	-	-	+	+	-	-	-	-	-	-	-	-
*Frigoribacterium* (Actinobacteria)	-	-	-	-	-	-	-	-	-	-	-	+	-	-	-	-	-	-	-	-
*Janthinobacterium lividum* (Betaproteobacteria)	-	-	-	-	+	+	-	-	-	-	-	-	-	-	-	-	+	-	-	-
*Leifsonia poae* (Actinobacteria)	-	-	-	-	-	-	-	-	-	-	-	-	-	-	-	-	-	+	-	-
*Massilia timonae* (Betaproteobacteria)	-	-	-	-	-	-	-	-	-	-	-	-	+	-	-	-	-	-	-	-
*Methylobacterium* (Alphaproteobacteria)	-	-	-	-	-	-	-	-	+	-	-	-	-	-	-	-	-	-	-	-
*Microbacterium* (Actinobacteria)	-	-	-	+	-	-	-	-	+	-	-	+	-	-	-	+	-	-	-	-
*Mycetocola* (Actinobacteria)	-	-	-	-	-	-	-	-	-	-	-	-	-	-	-	-	+	-	-	-
*Paenibacillus* (Firmicutes)	-	-	-	-	-	-	-	-	-	-	-	-	-	-	+	+	-	-	-	-
*Pantoea* (Gammaproteobacteria)	-	-	-	+	+	-	-	+	+	-	-	-	+	-	+	-	+	+	-	-
*Pedobacter* (Bacteroidetes)	-	-	-	-	-	-	-	-	-	-	-	-	-	+	-	-	-	+	-	-
*Pseudomonas fluorescens* (Gammaproteobacteria)	+	-	-	-	+	+	-	-	+	-	+	+	+	-	-	-	-	-	-	+
*Pseudomonas chloroaphis* (Gammaproteobacteria)	+	-	+	+	-	-	-	-	-	-	-	-	-	-	-	-	-	-	-	-
*Pseudomonas syringae* (Gammaproteobacteria)	-	-	-	-	+	+	-	-	-	-	-	+	-	-	-	-	-	-	-	+
*Pseudomonas* (other) (Gammaproteobacteria)	+	+	+	-	+	+	+	+	+	+	+	+	+	-	+	-	+	+	+	+
*Serratia* (Gammaproteobacteria)	-	-	-	-	-	-	-	-	-	-	-	-	-	-	-	-	-	-	+	+
*Shewanella* (Gammaproteobacteria)	-	-	+	-	-	-	-	-	-	-	-	-	-	-	-	-	-	-	-	-
*Sphingobacterium* (Bacteroidetes)	-	-	-	-	-	-	-	-	-	-	-	-	-	+	-	-	+	+	-	-
*Sphingobium* (Alphaproteobacteria)	-	-	-	-	-	-	-	+	-	-	-	-	-	+	-	-	-	-	-	-
*Sphingomonas* (Alphaproteobacteria)	-	-	-	-	-	-	-	-	+	-	-	-	-	-	-	-	-	-	-	-
*Stenotrophomonas* (Gammaproteobacteria)	-	-	-	-	-	-	+	-	-	-	-	-	-	-	+	-	+	-	+	-
*Xanthomonas* (Gammaproteobacteria)	-	-	-	-	+	-	-	-	-	-	-	-	+	-	-	-	-	+	-	-

Samples are baby spinach, romaine lettuce, red leaf lettuce, iceberg lettuce, and green leaf lettuce in conventional (C) and organic (O) varieties. Each was also subject to surface sterilization (designated by an s) to examine just the endophytic community. + indicates if an isolate of that taxa was obtained from a specific sample.

Other taxa were isolated from 20% or less of the samples plated (i.e. from just one to four samples) and included various genera that are known plant pathogens (e.g. *Agrobacterium*, *Erwinia*, *Leifsonia poae*, *Xanthomonas*) or non-pathogenic symbionts (e.g. *Curtobacterium*, *Massilia*, *Methylobacterium*, *Serratia*, *Stenotrophomonas*) [5,20]. As with *Pantoea*, these taxa are likely to be specific plant-associated strains, although some of these lineages (e.g. *Massilia timonae*, *Serratia*, *Stenotrophomonas*) can include potential human pathogens. Other culturable bacteria are probably also present in these samples, given that our isolation strategy focused only on the numerically dominant colonies (i.e. those growing on plates from the greatest dilution), and only on those that appeared morphologically distinct. Use of additional media types may also have led to a greater number of distinct isolates, although the two types of growth medium used represent both a rich, general purpose media (TSA) and one more commonly used on nutrient poor environmental samples (R2A agar) [24]. That said, while approximately half of the isolates were obtained on R2A agar, all of them were capable of growth on TSA and this medium was eventually used for the maintenance of all cultures.

### Culture independent analyses

**Figure 2:**
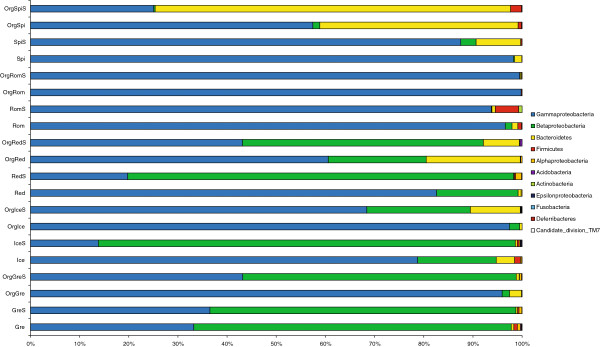
**Relative abundance of bacterial phyla associated with leafy salad vegetables as determined from pyrosequencing.** Samples are organically (Org) and conventionally grown baby spinach (Spi), romaine lettuce (Rom), red leaf lettuce (Red), iceberg lettuce (Ice), and green leaf lettuce (Gre) and include intact and surface sterilized (S) subsamples. Percentages represent the portion of 16S rRNA gene 454 reads (mean 2,515 per sample) that were classified to that phylum (or subphylum in the case of Proteobacteria).

A total of 50,339 non-chimeric partial 16S rRNA gene sequences of >200 bp were obtained from community DNA 454 pyrosequencing. With the use of primers designed to avoid chloroplasts, just 24 of these sequences proved to be chloroplast derived and an additional 16 could not be grouped to any recognized bacterial phylum, leaving 50,299 for subsequent analyses, or a mean of 2,515 per sample. Across all samples, a total of 634 OTUs were detected, representing 11 different bacterial phyla (or subphyla in the case of the Proteobacteria; Figure 2). Gammaproteobacteria and Betaproteobacteria were the dominant lineages in almost all leaf vegetable samples, regardless of surface sterilization or agricultural type, and accounted for at least 90% of the sequences obtained in all but three samples (Figure 2). Exceptions were the sample of unsterilized organically grown red leaf lettuce (from which they accounted for 80% sequences obtained), and the samples of both unsterilized and surface sterilized organically grown baby spinach (from which they accounted for 59% and 25% of the sequences, respectively). In each of these three cases, sequences from the phylum Bacteroidetes were more prevalent, and were actually the most prevalent lineage detected in the surface sterilized organically grown baby spinach (Figure 2). Other major bacterial lineages that were prevalent in multiple samples were the Firmicutes, Alphaproteobacteria, Acidobacteria, and Actinobacteria, although each of these lineages accounted for an average of less than 1% of the sequences obtained. Sequences affiliated with the Epsilonproteobacteria (surface sterilized conventional iceberg lettuce), Fusobacteria (surface sterilized organic iceberg lettuce), Deferribacteres (surface sterilized organic baby spinach), and candidate division TM7 (conventional green leaf lettuce) were detected in very low amounts in just one sample each. By comparison, Rastogi et al. [25] found that Proteobacteria, Firmicutes, and Bacteroidetes were the most abundant phyla in the romaine lettuce phyllosphere, and Lopez-Velasco et al. [26] found that Proteobacteria and Firmicutes were the dominant phyla in the phyllosphere of spinach. As in this study, Gammaproteobacteria were recently reported as the most prevalent lineage present on the surface of a variety of produce types [19], and were primarily identified as members of the Enterobacteriaceae.

At a finer taxonomic level, 23 different taxa were identified that accounted for > 0.1% of the sequences detected across all samples (i.e. taxa that composed at least 1/1000 of the sequences analysed; Table 2). Definitive identification to the species level was not possible given the short sequence length (mean 210 bp), but identification to genus was generally possible. *Pseudomonas* (Gammaproteobacteria) was the most prevalent genus in eight of the 20 samples, and has been reported by others to be the most prevalent genus in the phyllosphere of spinach and lettuce when analysed by culture-independent techniques [25-27]. *Ralstonia* (Betaproteobacteria) was the most numerous genus in six samples (five of which were surface sterilized), *Xanthomonas* (Gammaproteobacteria) in two (non-sterilized conventionally grown romaine and iceberg lettuce), and *Flavobacterium* (Bacteroidetes), *Stenotrophomonas* (Gammaproteobacteria), *Serratia* (Gammaproteobacteria), and *Erwinia* (Gammaproteobacteria) in one each (sterilized organic baby spinach, sterilized organic romaine lettuce, non-sterilized organic green leaf lettuce, and non-sterilized organic iceberg lettuce, respectively). Taxa identified by this culture-independent approach included widely recognized plant pathogens or symbionts (e.g. species of *Pseudomonas*, *Ralstonia*, *Stenotrophomonas*, *Erwinia*, *Xanthomonas*, *Janthinobacterium*, *Massilia*, *Chryseobacterium*), but also some genera that contain species that are potential human pathogens (e.g. *Pseudomonas*, *Serratia*, *Providencia*, *Enterobacter*, *Morganella*, *Bacillus*, *Streptococcus*, *Staphylococcus*). Only two taxa showed statistically significant (p < 0.05) differences in their prevalence when all vegetable types were compared in terms of organic versus conventionally grown, or non-sterilized versus surface sterilized. *Ralstonia* accounted for a significantly greater proportion of the bacterial community in the surface sterilized samples, implying that it was largely endophytic, and was also a significantly lower proportion of the community in the samples from organically grown varieties. *Acinetobacter* accounted for a significantly lower proportion of the community in surface sterilized samples, suggesting that it was primarily associated with the leaf surface.

**Table 2:** Dominant members of bacterial communities associated with leafy salad vegetables as determined from pyrosequencing

**Genus (or higher)**	**Baby spinach**				**Romaine lettuce**				**Red leaf lettuce**				**Iceberg lettuce**				**Green leaf lettuce**			
	**C**	**Cs**	**O**	**Os**	**C**	**Cs**	**O**	**Os**	**C**	**Cs**	**O**	**Os**	**C**	**Cs**	**O**	**Os**	**C**	**Cs**	**O**	**Os**
*Pseudomonas*	93.8	70.6	40.5	20.7	23.9	67.0	67.2	36.1	76.3	18.9	54.7	27.4	11.1	7.1	2.5	59.9	28.7	33.2	5.1	15.0
*Ralstonia* *(S, O)	-	-	-	-	-	-	-	-	11.8	76.5	1.6	38.7	14.7	82.7	0.7	20.4	60.7	60.3	-	53.4
*Flavobacterium*	1.5	8.9	38.9	72.1	1.1	0.5	-	0.3	0.2	0.1	18.5	7.3	3.6	0.3	-	9.4	0.3	0.1	2.0	0.5
*Stenotrophomonas*	-	2.3	0.1	2.8	20.2	20.0	30.8	62.2	-	0.1	-	0.2	1.9	0.5	1.0	1.3	0.5	1.1	-	0.3
*Serratia*	1.2	0.2	-	0.1	-	-	-	-	-	-	0.1	1.3	5.1	3.7	-	0.7	0.3	-	66.0	18.6
*Erwinia*	1.9	10.5	-	0.1	0.2	-	0.1	-	0.1	-	-	-	1.3	0.2	58.6	0.8	0.3	-	0.4	0.1
*Xanthomonas*	-	-	-	-	47.4	-	0.1	-	-	-	-	-	51.4	0.5	-	-	-	-	-	-
*Pantoea*	0.1	1.4	0.1	0.1	1.0	3.0	-	0.1	0.1	0.1	-	0.1	1.1	0.1	17.6	1.1	1.1	0.6	0.1	0.3
*Providencia*	-	-	-	-	-	-	-	-	-	-	-	0.1	0.8	0.5	-	-	-	-	13.9	3.9
Enterobacteriaceae unk..	0.8	0.9	1.0	0.2	2.1	0.5	0.7	0.4	0.3	0.1	1.3	0.4	2.1	0.3	0.5	0.6	0.6	0.2	0.8	1.8
*Janthinobacterium*	0.2	2.9	1.2	0.2	0.4	-	-	-	0.1	-	7.6	4.1	0.3	0.2	-	0.3	0.3	0.1	0.8	0.5
*Shewanella*	-	-	13.1	0.4	-	-	0.1	-	-	-	-	-	-	-	-	-	-	-	-	-
*Enterobacter*	0.1	0.2	-	0.3	-	0.4	-	-	0.5	0.3	-	0.5	1.4	0.6	2.4	-	-	-	2.6	1.3
*Enhydrobacter*	-	-	-	-	0.1	-	-	-	2.3	-	3.4	3.5	0.1	-	-	-	-	-	0.3	0.3
*Leeia*	-	-	-	-	-	-	-	-	1.2	1.0	-	1.5	0.1	0.5	-	1.3	1.3	0.9	-	0.8
*Morganella*	-	-	-	-	-	-	-	-	-	-	-	8.5	-	-	-	-	-	-	-	-
*Massilia* *(S)	-	-	0.1	0.1	0.2	-	-	-	1.3	-	1.7	1.3	0.4	0.1	-	0.2	0.2	0.1	0.2	0.1
*Duganella*	0.1	-	-	-	-	-	-	-	0.4	-	3.5	0.9	0.1	-	0.2	0.1	0.1	-	-	-
*Acinetobacter* *(S)	-	-	0.8	-	0.2	-	-	-	0.1	-	0.5	0.1	0.5	-	0.4	0.1	-	-	0.6	0.2
*Bacillus*	-	-	-	-	-	3.4	-	-	-	0.2	-	-	-	-	-	-	-	-	-	-
*Streptococcus*	-	-	0.2	1.5	0.1	0.1	-	-	-	-	-	-	-	0.4	-	-	0.1	0.1	-	-
*Staphylococcus*	-	-	0.3	0.4	0.3	0.1	-	-	-	-	-	-	1.1	-	-	-	0.5	-	-	-
*Chryseobacterium*	-	0.2	0.9	-	-	0.2	-	-	-	-	0.2	-	0.1	-	0.4	-	-	-	0.2	-

Samples are baby spinach, romaine lettuce, red leaf lettuce, iceberg lettuce, and green leaf lettuce in conventional (C) and organic (O) varieties. Each was also subject to surface sterilization (designated by an s) to determine just the endophytic community. Numbers are the % of the total number of sequences (mean 2,515 per sample) for each sample that were classified as a particular taxa, and only taxa accounting for > 0.1% of the sequences across all samples are shown. *indicates taxa that accounted for significantly different (p < 0.05) percentages of the total community between either sterilized and non-sterilized samples (S) or conventional versus organic production (O).

While sequences corresponding to 23 taxa were detected at a frequency that was > 0.1% of all of the sequences examined, other “rare” OTUs were detected at low levels. Of the 634 different OTUs recognized, 319 were represented by just one sequence read in a single sample, and a further 104 by just two sequence reads. The number of OTUs detected in each sample, when standardized to the same number of reads, was used as a simple measure of bacterial community diversity. An average of 47 OTUs were detected in each sample, but this varied from 17 (the samples from surface-sterilized and non-sterilized organic romaine lettuce) to 92 (the organic red leaf lettuce sample; Table 3). These values are in the same range as those reported for the leaf surface bacterial communities on store-bought lettuce and spinach [19], and are similar or slightly lower than diversity estimates reported for stems and leaves of alfalfa [3]. However, they are an order of magnitude lower than estimates of bacterial endophyte diversity derived from pyrosequencing of potato roots [2], although that study relied on diversity statistics (e.g. the Chao statistic) rather than directly assessing the number of distinct OTUs. Bacterial densities in leaves are also thought to be lower than those in roots or the rhizosphere [5,20], which may account for less diverse bacterial communities in above-ground plant structures. There were no consistent patterns in OTU richness in regards to organic versus conventional produce or in terms of surface-sterilized versus non-sterilized samples (p > 0.05 for both comparisons), but surface-sterilized (i.e. endophyte) diversity was moderately correlated with overall bacterial diversity determined from the non-sterilized samples (R = 0.68). It should be noted, that these diversity estimates are likely to be low given that sequences were grouped into OTUs based on the more conservative 97% similarity criterion and that rarefaction curves (Additional file 1) did not always reach an asymptote.

**Table 3:** Diversity statistics for bacterial communities associated with leafy salad vegetables as determined from pyrosequencing

**Diversity statistics**	**Baby spinach**				**Romaine lettuce**				**Red leaf lettuce**				**Iceberg lettuce**				**Green leaf lettuce**			
	**C**	**Cs**	**O**	**Os**	**C**	**Cs**	**O**	**Os**	**C**	**Cs**	**O**	**Os**	**C**	**Cs**	**O**	**Os**	**C**	**Cs**	**O**	**Os**
Number of OTUs	22	34	57	31	46	42	17	17	71	40	92	73	54	48	36	48	62	41	52	64
Total sequence reads obtained	5356	2142	2333	2610	2347	1769	2716	2702	3073	2113	2155	1640	1507	2633	2808	2740	2371	2423	2512	2365

Samples are baby spinach, romaine lettuce, red leaf lettuce, iceberg lettuce, and green leaf lettuce in conventional (C) and organic (O) varieties. Each was also subject to surface sterilization (designated by an s) to determine just the endophytic community. Values are derived from a standardized 1,507 OTU sequences per sample.

**Figure 3:**
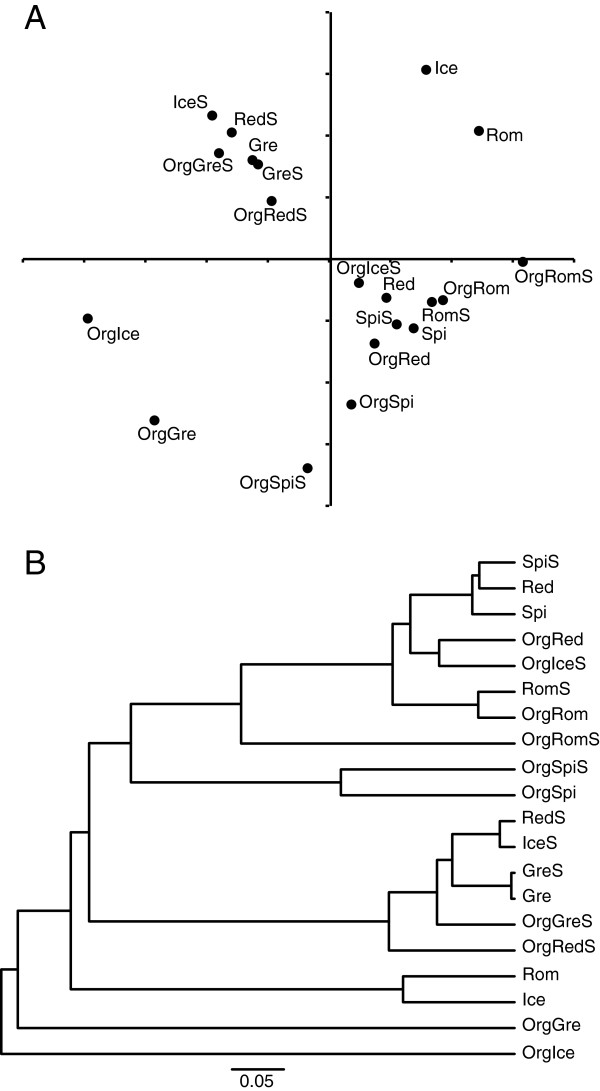
**Similarities of bacterial communities associated with leafy salad vegetables as derived from pyrosequencing.** Samples are organically (Org) and conventionally grown baby spinach (Spi), romaine lettuce (Rom), red leaf lettuce (Red), iceberg lettuce (Ice), and green leaf lettuce (Gre) and include intact and surface sterilized (S) subsamples. Community similarity is determined from Jaccard similarity scores followed by nonmetric multidimensional scaling **(A)** or UPGMA dendrogram construction **(B)**. Analyses are run on subsamples of 1507 sequences from each sample, and show the mean outcome of 1000 individual subsampling runs.

NMDS was used to ordinate each sample in order to evaluate community similarity, i.e. to determine if similar endophytic or overall bacterial populations were associated with the different leaf vegetables or sampling treatments. Two dimensional NMDS based on theta dissimilarity scores was sufficient to account for community differences (stress = 0.19, r^2^ = 0.81), but yielded few consistent patterns in regards to vegetable type, surface sterilization, and organic or conventional production (Figure 3A). AMOVA confirmed this, with there being no statistically significant differences between samples based on groupings of organic versus conventional (p = 0.17), or surface sterilized versus non-sterilized (p = 0.23). Date of sample purchase was likewise not related to community composition (p = 0.38). Vegetable type did result in significantly different groupings of samples (p = 0.006), however no individual comparisons between pairs of salad vegetable types were significant following the Bonferroni correction (p > 0.005 for all). This pattern based on salad vegetable type was largely driven by the bacterial community associated with the samples of romaine lettuce, which while not statistically significantly different from that on any other individual lettuce type, had a low probability of occurring by chance (p = 0.016-0.049 for the various comparisons). The dendrogram of community similarity (Figure 3B) also showed no consistent separation of endophyte (surface sterilized) assemblages from overall plant associated bacterial communities, a finding that was confirmed by the UniFrac analysis (D = 0.69, p = 0.516). The UniFrac metric did suggest a marginally significant difference between organic and conventionally grown samples (D = 0.79, p = 0.04), but no overall effect of lettuce type (pairwise D scores 0.70-0.84, p > 0.10 for all). A survey of native plants on a prairie reserve found that host plant species did have a significant effect on the leaf endophyte community [28], although that study examined five quite different plant species, rather than the five similar varieties of salad vegetables sampled in this study. Different types of produce ranging from mushrooms to apples have been found to have distinct bacterial communities on their surface, although certain produce types (e.g. spinach, lettuce, sprouts) may have more similar phyllosphere communities [19], as reported here.

### Comparing the culture dependent and culture independent approaches

A paradigm in microbial ecology is that culture-based techniques only recover 1-10% of the true bacterial diversity within an environment [29,30] and that molecular surveys of bacterial communities yield dramatically different results than traditional culture approaches. Comparing the number of different isolated bacterial species (31 total) obtained in this study to the overall number of OTUs (634 total) obtained from pyrosequencing would initially seem to confirm this concept. However, many of the proportionally dominant taxa identified by the pyrosequencing approach were actually represented by isolates (Tables 2 and 3). A similar outcome has been reported for *Arabidopsis thaliana*, in that many of the endophytic populations detected by pyrosequencing were related to culturable species [31]. In the current study, *Pseudomonas* spp. were the most prevalent taxa in the majority of samples according to the molecular approach, and strains of *Pseudomonas* were isolated from all but two samples (surface sterilized iceberg lettuce). Other taxa that were proportionally dominant in some samples according to community sequencing included *Flavobacterium*, *Stenotrophomonas*, *Serratia*, *Erwinia*, *Xanthomonas*, and *Pantoea*; all of which were also obtained as isolates, often from samples that showed higher proportions of that taxa in the sequence collection. Our culture approach was by no means exhaustive (just two media types, and only selecting colonies that appeared to be abundant based on morphology), suggesting that compared to other environmental samples it may be relatively easy to isolate the more dominant members of some plant-associated bacterial communities, or at least those associated with salad produce. A notable exception was *Ralstonia* which, while absent from nine samples, was the most abundant sequence type detected in six samples but was not obtained as an isolate. Species of *Ralstonia* are typically capable of growth on TSA, but colonies are commonly small [32] so may have not been chosen during our isolate selection. *Ralstonia* was, however, one of the few taxa to show significant differences between samples, being present in greater proportions in surface sterilized and/or conventionally grown samples. It’s omission from detection by the culture dependent procedure meant that solely using that approach would have missed one of the few patterns in the distribution of bacterial populations between these samples. While the pyrosequencing approach yielded much greater diversity estimates, much of that diversity came from OTUs that were present as low numbers of sequence reads in few samples, and these are unlikely to represent major endophytic or phyllosphere populations.

### Broader implications

The broader public is likely unaware that most, if not all, plant species contain endophytic populations. While the vast majority of endophytes are likely to be harmless to a typical consumer, internalization of pathogens within produce is a critical issue as these internalized, endophytic bacteria have essentially no chance of being removed from salad produce during post-harvest or consumer processing [33]. Based on the enumeration of culturable bacteria from surface sterilized produce in the current study, consumers could be consuming up to 4.9 × 10^7^ endophytic bacteria in a typical serving (approximately 85 g) of salad, even if all surface-associated bacteria could be removed by aggressive washing and surface sterilization techniques. A more typical pre-consumption washing procedure would result in the consumption almost 100× more bacteria (4.7 × 10^9^) in a salad serving, a mixture of endophytes and surface-associated cells. As such, enumerating and identifying the microbial community within minimally processed plant crops is of potential concern from a health safety standpoint, either for the direct detection of internalized pathogens, or because some native endophytic populations may serve as antagonists to pathogen growth and survival.

Molecular studies of the phyllosphere and endophytes have lagged behind those of soils and waters. Traditionally, studies of plant-associated bacteria have used culture-based methods, although culture-independent methods to analyse endophyte and phyllosphere bacterial diversity are now being utilized with greater frequency e.g. [27,28,34,35]. Pyrosequencing has begun to be employed to investigate plant-associated bacterial communities, such as those colonizing the roots and leaves of *Arabidopsis thaliana*[31,36,37], and phyllosphere populations on the surface of various leaves [18,25,26,38]. Studies of bacterial communities in vegetable produce at the time of consumption are much less common, a recent exception being the study by Leff and Fierer [19], who used pyrosequencing to survey the bacteria associated with eleven produce types. However, even that study was limited to surface populations and did not address the presence of endophytes. Other studies have sampled immediately postharvest or during the growing period [25,26,38] and the bacterial communities in these plants may have changed over the time period from harvesting to consumer purchase. Extended storage at 4°C has been found to decrease the richness and diversity of bacteria in the phyllosphere [26] although it is unclear what effect refrigerator storage has on the endophytic component of the microbial community. Regardless, analysis of store bought vegetables more truly represents what microorganisms are likely to be consumed by the typical consumer.

A recent study examining store bought lettuce found that 38 out of 100 leaves had internalized bacteria; although this conclusion was based solely on culture-dependent methods [39]. A few other studies have used pyrosequencing to analyse the phyllosphere bacterial community on lettuce and spinach [19,25,26], although those studies retrieved the phyllosphere community from washes from leaves and thus exclude endophytes, as well as any bacteria that adhere tightly to the leaf surface. We used a different approach, in which we surface-sterilized the surface, killing the bacterial populations associated with the leaf surface. Thus our non-sterilized samples include all leaf-associated populations (endophytes and surface-associated), while our surface sterilized samples represent just the endophytes. To our knowledge, the study presented here is the first report of pyrosequencing analysis of the endophytic bacterial community associated with store bought, ready-to-eat produce.

## Conclusions

Commercial ready-to-eat salad leaf vegetables harbor an array of endophytic and surface associated bacteria. Culture-independent analysis using pyrosequencing indicated that the majority of leaf vegetable-associated bacteria were members of the Proteobacteria and Bacteroidetes. Dominant bacterial taxa identified by pyrosequencing were also identified as culturable isolates. However, the use of pyrosequencing also allowed for the identification of numerous low abundance bacteria that would not have been identified otherwise by culture dependent methods. Whether vegetables were cultivated under conventional or organic agricultural systems appeared to have little consistent impact on the microbial community composition. While surface sterilization significantly decreased the number of bacteria, surface sterilized salad vegetables still contained at least 2.2 × 10^3^ to 5.8 × 10^5^ culturable endophytic cells per gram of leaf material. Even the most extreme washing would not remove these cells, so that consumers are constantly exposed to appreciable levels of plant-associated microorganisms.

## Methods

### Sample collection and processing

Packages of ready-to-eat leaf vegetables were purchased from a grocery store in Oxford, Mississippi, USA, during September and October 2010. Leaf vegetables consisted of romaine lettuce and baby spinach (both purchased September 15^th^ 2010), and green leaf lettuce, iceberg lettuce, and red leaf lettuce (all purchased October 11^th^ 2010). Both organic and conventionally grown varieties of each produce type were obtained (ten samples total). Samples were in modified atmosphere packaging, stored in the chilled produce section. All vegetable types were packaged as leaves or leaf pieces, with just a single type of vegetable per pack, and were labelled as “ready to eat” or similar. Samples were collected one day prior to laboratory procedures and stored overnight in a domestic refrigerator (5°C) prior to processing. For each sample, microbiological and molecular analyses were conducted on both intact (unsterilized) material and on surface sterilized material. Unsterilized samples (an assortment of leaves corresponding to 10–20 g of leaf material) were washed under regular tap water (as might be done by a typical consumer) and then added to bottles containing 100 mL of sterile magnesium phosphate buffer [40].

Surface sterilized samples (10–20 g of leaf material) were washed in the same manner as unsterilized samples and then placed into sterile sample bottles. These bottles then received 100 ml of a 1.3% sodium hypochlorite solution and were shaken (200 rpm) for 5 min. The sodium hypochlorite solution was decanted and replaced with 70% ethanol, and bottles were shaken for a further 2 min. The ethanol was decanted, replaced with 100 ml sterilized distilled water, and bottles were shaken for 10 seconds. The water was removed and this sterile water rinse repeated three more times to ensure that there was minimal sodium hypochlorite or ethanol remaining in the bottle. Following the final wash, 100 mL of sterile magnesium phosphate buffer was added to the bottle. Efficiency of this sterilization technique was tested by wiping of sterilized leaves of each type across the surface of a trypticase soy agar (TSA) plate, which consistently yielded no bacterial colonies.

### Culture dependent microbiological analyses

Surfaced sterilized and unsterilized samples were homogenized using a Power Gen 500 homogenizer (Fisher Scientific) and the resulting leaf slurries serially diluted ten-fold. Subsamples (0.1 mL) of each dilution were plated in triplicate onto both TSA and R2A agar; each medium also contained 0.1 g L^-1^ cycloheximide to inhibit fungal growth. Plates were incubated at room temperature (22°C) for 2–5 d, after which time colonies were counted and final counts expressed as CFU g^-1^ leaf vegetable.

Colonies were qualitatively typed based on color and overall morphology, and a sample of each numerically dominant morphological colony type was transferred onto a new plate of the appropriate medium and incubated (22°C; 2–4 d). These isolates were transferred three times to ensure purity. Following growth of the third transfer, DNA was extracted from a single colony of each isolate using UltraClean Microbial DNA Isolation kits (Mo Bio Laboratories, Carlsbad, CA). A portion of the 16S rRNA gene was amplified using the Bac799f and Univ1492r primers with amplification conditions described below and amplicons subsequently sequenced. Potentially erroneous bases (low quality scores) were removed and sequences were then processed through the Greengenes database [41] in order to identify and classify them.

### Culture independent molecular analyses

Following homogenization of each sample, a subsample of the slurry (50 mL) was filtered through a sterile 11 Whatman 1 filter (11 μm nominal pore size) to remove residual leaf particles. 35 mL of the filtrate was collected and centrifuged (8,000 × g, 10 min) to pellet cells, and the moist pellet transferred to a 1.5 ml sterile microcentrifuge tube. This pellet was further centrifuged (8,000 × g, 10 min), the supernatant removed, and the pellet frozen at −20°C until DNA extraction. DNA was extracted using a PowerSoil DNA Isolation Kit (Mo Bio Laboratories, Carlsbad, CA) and a fragment of the bacterial 16S rRNA gene amplified using Bac799f (5’-AACMGGATTAGATACCCKG-3’) and Univ1492r (5’-GGTTACCTTGTTACGACTT-3’) primers. This combination of primers targets bacterial DNA specifically without amplifying residual chloroplast DNA from the host plant. Plant mitochondrial DNA is co-amplified, but yields a 1,090 bp fragment compared to a 735 bp fragment for bacterial DNA [42-44]. PCR was carried out in 50 μl reactions following procedures described previously [44]. Amplification products were visualized on 1% agarose gels, which also separated bacterial and host plant mitochondrial DNA fragments. The bacterial gel band was excised and DNA recovered from the gel fragments using UltraClean GelSpin DNA Extraction Kits (Mo Bio Laboratories, Carlsbad, CA). These purified bacterial 16S rRNA gene fragments were used as the templates for pyrosequencing. Negative control amplifications (no template DNA) were carried out routinely and yielded no detectable product.

Bacterial tag-encoded FLX amplicon 454 pyrosequencing (bTEFAP) [45] was conducted on the 16S rRNA gene amplicons of each sample, through a dedicated sequencing facility (MR DNA, Shallowater, TX). Bacterial primers 939f and 1392r [46,47] were used in the sequencing reaction. A single-step PCR using HotStarTaq Plus Master Mix Kit (Qiagen, Valencia, CA) was used under the following conditions: 94°C for 3 min, followed by 28 cycles of 94°C for 30 sec, 53°C for 40 sec, and 72°C for 1 min, after which a final elongation step at 72°C for 5 min was performed. Following PCR, all amplicon products from different samples were mixed in equal concentrations and purified using Agencourt AMPure XP beads (Agencourt Bioscience Corporation, Danvers, MA). Samples were sequenced utilizing Roche 454 FLX titanium instruments and reagents and following the manufacturer’s guidelines. A negative control amplification was used in the same 454 reaction and gave no valid reads.

Raw pyrosequence data derived from the sequencing process was transferred into FASTA files for each sample, along with sequencing quality files. Files were accessed using the bioinformatics software Mothur [48] where they were processed and analysed following general procedures recommended by Schloss et al. [49]. Briefly, sequences were denoised, and trimmed to remove barcodes and primers. Sequences were aligned and classified according to those in the SILVA rRNA database [50], after which chimeric sequences and any sequences classified as mitochondria or chloroplasts were removed from the dataset. Remaining sequences were grouped into operational taxonomic units (OTUs) based on a 97% similarity criterion. Rarefaction was performed on each sample to assess sampling adequacy, using a 50 sequence increment. Random subsamples (1000) of OTUs from each sample corresponding to the number of sequences in the lowest sample (i.e. smallest sample size) were then used for further analysis. The same subsampling approach was used to examine variation in community structure between samples (beta diversity) using the theta similarity index of Yue and Clayton, an index that accounts for proportional abundances of both shared and non-shared OTUs [51]. Similarity between samples was visualized by ordination of samples by non-metric multidimensional scaling (NMDS) as well as dendrogram construction. Spatial separation of samples in NMDS was tested through analysis of molecular variance (AMOVA), while clustering of samples within the dendrogram was tested using the UniFrac distance metric [52].

### Availability of supporting data

All sequences used in this study are available in the NCBI Sequence Read Archive under study accession SRP032750 (http://www.ncbi.nlm.nih.gov/Traces/sra/sra.cgi?study=SRP032750).

## Competing interests

The authors declare that they have no competing interests.

## Authors’ contributions

CRJ conceived of the study, conducted the bioinformatics and statistical analyses and drafted the manuscript. KCR and SLO carried out the sample processing, culture dependent analyses, and initial molecular work. HLT carried out amplifications for pyrosequencing, later molecular work, and assisted with manuscript preparation. All authors read and approved the final manuscript.

## Supplementary Material

Additional file 1 **Rarefaction of pyrosequencing data.** Rarefaction analysis of the 454 pyrosequencing data for each sample as performed in mothur using the “rarefaction” command, with a 50 read increment. Click here for file
